# Clinical and Genetic Correlation in Neurocristopathies: Bridging a Precision Medicine Gap

**DOI:** 10.3390/jcm13082223

**Published:** 2024-04-11

**Authors:** Despoina Chatzi, Stella Aikaterini Kyriakoudi, Iasonas Dermitzakis, Maria Eleni Manthou, Soultana Meditskou, Paschalis Theotokis

**Affiliations:** Department of Histology-Embryology, School of Medicine, Aristotle University of Thessaloniki, 54124 Thessaloniki, Greece; chatzidc@auth.gr (D.C.); kstellaai@auth.gr (S.A.K.); iasonasd@auth.gr (I.D.); mmanthou@auth.gr (M.E.M.); sefthym@auth.gr (S.M.)

**Keywords:** neural crest, neurocristopathies, congenital anomalies, cancers, rare diseases, precision medicine

## Abstract

Neurocristopathies (NCPs) encompass a spectrum of disorders arising from issues during the formation and migration of neural crest cells (NCCs). NCCs undergo epithelial–mesenchymal transition (EMT) and upon key developmental gene deregulation, fetuses and neonates are prone to exhibit diverse manifestations depending on the affected area. These conditions are generally rare and often have a genetic basis, with many following Mendelian inheritance patterns, thus making them perfect candidates for precision medicine. Examples include cranial NCPs, like Goldenhar syndrome and Axenfeld–Rieger syndrome; cardiac–vagal NCPs, such as DiGeorge syndrome; truncal NCPs, like congenital central hypoventilation syndrome and Waardenburg syndrome; and enteric NCPs, such as Hirschsprung disease. Additionally, NCCs’ migratory and differentiating nature makes their derivatives prone to tumors, with various cancer types categorized based on their NCC origin. Representative examples include schwannomas and pheochromocytomas. This review summarizes current knowledge of diseases arising from defects in NCCs’ specification and highlights the potential of precision medicine to remedy a clinical phenotype by targeting the genotype, particularly important given that those affected are primarily infants and young children.

## 1. Introduction

In early embryonic development, the neural stage begins with the formation of the primitive neural plate, which subsequently folds to form the neural tube, the precursor to the central nervous system. Specific molecular cues and drivers strictly regulate each developmental process [1,2]. The neural crest arises on each side of the neural plate between the neural and non-neural ectoderm [3,4]. Comprising cephalic, cardiac, truncal, and enteric regions, the neural crest contributes to various tissues and systems, including head and neck structures, the heart outflow tract, pigment cells, peripheral nervous system, and enteric nervous system [5]. Before commencing migration, NCCs undergo epithelial–mesenchymal transition (EMT). During EMT, epithelial cells undergo structural and molecular changes, including the loss of junctions and polarity and cytoskeleton reorganization, ultimately enhancing cell motility and promoting the mesenchymal migratory capacity [6].

The broad spectrum of cells originating from the neural crest can result in various pathologies known as neurocristopathies (NCPs), impacting multiple systems [7]. Additionally, deregulated EMT plays a role in advancing tumor growth and facilitating metastasis [8]. Robert Bolande (1974) pioneered the conceptual framework for NCPs and its initial nomenclature [7]. Bolande grossly categorized NCPs into distinct, tumor-based classes and syndromes. However, NCPs do not conform strictly to such delineations, as evidenced by a significant intersection across developmental stages in tissue and organ formation, where these conditions manifest [9]. Research exploring the developmental signaling pathways governing NCC migration and differentiation has revealed pivotal molecular components that undergo dysregulation in NCPs.

Despite genetic screening being the primary method for detecting and diagnosing genetic disorders, the identification of causative genes remains limited, encompassing only a minority of patients. Therefore, there is a concerted effort to unveil additional genes implicated in typical development perturbations, with intensive investigations employing various animal models underway. Assessing the functionality of these candidate genes in animal models, which closely resemble humans, holds promise for promptly identifying key candidates, ensuring diagnostic accuracy, and facilitating timely intervention. In this review, initially, the pool of genes implicated before and after EMT are highlighted. Secondly, the NCPs listed here are categorized based on the origin of the problem within the neural crest, although there are numerous other disease processes that can involve multiple aspects of neural crest development. Lastly, NCP-related cancers are discussed.

## 2. Genes Involved in EMT during Neural Crest Development

### 2.1. Pre-EMT Genes

The induction of the neural crest requires the combined distribution of BMP, FGFs, and WNT inhibitors. When NCCs are specified, they undergo changes in gene regulation that ultimately steer their differentiation into specific cell types. As mentioned earlier, signals from BMP, FGF, WNT, and retinoic acid play a crucial role in inducing neural crest identity [3,10]. Specifically, FGFs initiate this process through the activation of proneural genes in the neural ectoderm, while BMP and WNT signaling molecules are situated at the interference of neural and non-neural ectoderm and their role is to stimulate several transcription factors, commonly referred to as specifiers of the neural plate border. Signals derived from BMP and WNT molecules are predominant in lateral areas of the developing embryo, whereas their suppressors, such as *Dkk* and *Cer* [11], conquer the central regions, thus establishing a mediolateral gradient so that the cells destined to become the neural plate border emerge from an area exposed to moderate levels of WNT and BMP activity, finally forming the neural fold [12]. The action of such signaling molecules leads to the stimulation of neural plate border genes that include *Msx1*, *Zic1*, *Tfap2*, *Pax3/7*, *Gbx2*, *Hairy2*, *Foxi1/2*, *Dlx5/6* and *Gata2/3* [10,13]. Notably, certain border specifier genes such as *Gbx2*, *Pax3*, and *Msx1*, appear to be influenced by Wnt signaling. The activation of *Pax3* might be indirect, as WNT signals also trigger the expression of *Tfap2*, which then directly binds to regulatory elements of *Pax3* [14,15]. Research conducted across various vertebrates has demonstrated that once these transcription factors start being expressed in the neural plate border region, they initiate a sequence of reciprocal regulatory interactions. These interactions result in the consolidation of this regulatory condition and guarantee the sustained expression of these factors [3]. The relatively slow development rate of the lamprey embryo makes it a useful tool in the investigation and apprehension of interactions between these early expressed genes, which can coactivate each other. For example, in zebrafish embryos, *tfap2* appears to play a crucial role in the activation of neural plate border specification and is essential for the activation of *gata2/3* and *foxi* genes [16,17]. Ultimately, the demarcation line separating the neural plate from the neural plate border is refined through inhibitory exchanges involving transcription factors of both neural and non-neural origins.

Moving further from the neural plate border, the first genes found to be expressed in chicks’ neural crests are Ets1, FoxD3 and Snai1/2 [13]. During gastrulation, Pax7 is linked to groups of cells that eventually give rise to the neural crest. Although not only neural crest progenitors express this gene, Pax7 is present in the cells that will become the neural crest, so we can conclude that it might restrict them to this fate [18]. Additionally, along with Pax3 and Msx1, it is necessary for the activation of Ets1 [19]. The combined activity of Zic1 and Pax3/7 in chicks controls the expression of FoxD3 in the developing trunk, with WNT signaling playing a crucial role in the expression of Snai2 and FoxD3, while in frog embryos, this combination is involved in the specification of the neural crest [20]. In Xenopus, WNT collaborates with pax3 and zic1 to kickstart differentiation [3].

Apart from FoxD3, Sip1/Zeb2 and Sox9/10 are considered premigratory transcription factors, usually cooperating with Twist and Snail1/2 in order to decrease the expression of Cadherin-6B and other epithelial cadherins and upregulate the transcription of more mesenchymal cadherins. Along with other neural crest markers, *TUBB3*, which produces an element of microtubules, is also expressed in premigratory cranial NCCs [21]. *Chd7*, which belongs to the chromodomain helicase-binding family [22], is expressed in Xenopus and mice in premigratory NCCs [23] and is present in neural crest-derived tissues in mice [24].

The expression of key EMT transcriptional regulators, such as *Snail2* or *Twist1*, is present. Wnt/β-catenin signaling has a direct impact on the expression of *Snail2*, which in turn directly inhibits the expression of E-cadherin and cadherin 6B. Additionally, this signaling pathway triggers the expression of mesenchymal cadherin 7 and cadherin 11 [25]. E-cadherin’s expression gradually decreases in the neural crest. In addition, *Twist* expression is under the control of the Hif signaling pathway, which also controls the expression of *CXCR4*, which encodes the receptor for the chemokine CXCL12/stromal cell-derived factor 1 [26].

**Figure 1:**
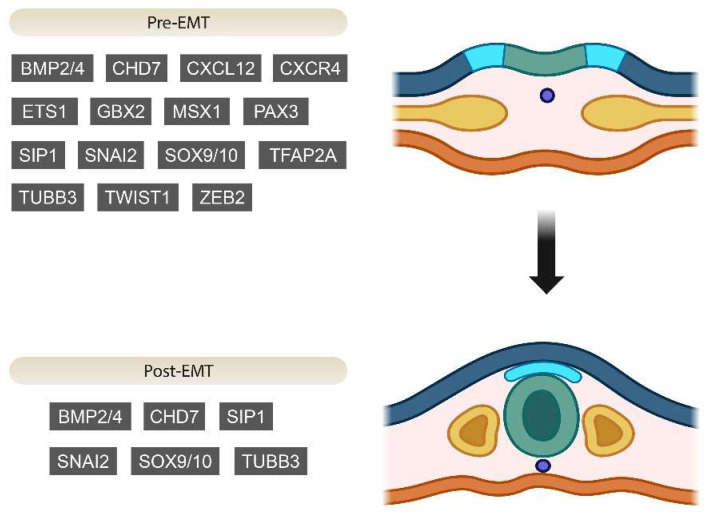
Genes involved concurrently in neurocristopathies and the epithelial–mesenchymal transition (EMT) of neural crest cells (NCCs).

The genes responsible for specifying neural crest development mutually enhance each other’s activity. For instance, in mice, *Sox10* expression is enhanced by *FoxD3*, while *foxD3*, *sox9*, and *twist* are upregulated under the influence of *snail2* in frog embryos. In this model, *snail2* is both self-activated and activated by *sox10* [3]. Pre-EMT genes implicated concurrently in NCPs and NCCs’ development during embryogenesis are presented in Figure 1.

### 2.2. Post-EMT Genes and Regulation

Research conducted across multiple species chickens, frogs, mice, and/or zebrafish), proves that EMT is partially regulated by the *Slug/Snail* family and *Sip1*, leading to changes in the cadherin expression of the cells in the mid-neurula stage. A prime example is *Sox9*, which together with *Slug/Snail* is enough to initiate the induction of EMT. *Foxd3*, *Slug/Snail*, and genes belonging to the SoxE family affect the levels of adhesion molecules that mediate cell-to-cell adhesion [26]. Research carried out in zebrafish provides knowledge of the downregulation of some genes, being related to the ability of NCCs to migrate. In this context, the Wnt target transcription factor Ovo1 inhibits genes related to the secretory pathway, such as *Rab3c*, *Rab12*, *Rab11fip2*, and *Sec6*, which all encode Rab GTPases [27]. *TUBB3*, which is also expressed before EMT, is present in early and late migrating NCCs, highlighting its connection to EMT and to the migration of NCCs [21]. *Chd7*, which belongs to the chromodomain helicase-binding family [22], is expressed in Xenopus and mice migratory NCCs [23] and is present later in neural crest-derived tissues in mice [24].

WNT signaling, also, plays a fundamental role in the delamination of NCCs. Canonical WNT signaling regulates the expression of cyclin D1 and facilitates the transition from the G1 phase to the S phase at the segmental plate mesoderm [28,29]. Additionally, BMP4 enhances the expression of its downstream gene *Wnt1*, leading to the phosphorylation of *Sox9*. This phosphorylation event, in turn, triggers an interaction with *Snail2*, promoting the delamination of NCCs in chick embryos. Furthermore, the BMP4/WNT pathway activates ADAM10, a protease protein, which cleaves the C-terminal fragment-1 of N-cadherin, which interacts with β-catenin [30]. This cleavage process produces CTF2, facilitated by γ-secretase, enhancing the transcriptional activity of β-catenin and cyclin D1. Consequently, this inhibits the G1/S transition, ultimately inducing the delamination of NCCs [31]. However, the connection between BMPs and WNT is more complex. In Xenopus and zebrafish, the ectoderm, from which the neural plate will develop, exhibits a BMP gradient. High BMP levels encourage ectoderm development, while low BMP levels support neural fate. Moderate BMP activity is thought to define the neural plate border and neural crest [32]. When it comes to Wnt, its levels were most elevated pre-EMT, and later, these levels significantly dropped after the NCCs delaminated. This might be in part due to activation of BMPs. There is reciprocal inhibitory communication between WNT and BMP signaling, where BMP hampers the proliferation induced by WNT, whereas WNT obstructs BMP-triggered neuronal differentiation [33].

Histone methylation is associated with the spatial and temporal regulation of gene expression in neural crest precursor cells [4]. The delay between the induction of NCCs during gastrulation and the initial activation of neural crest genes is linked to repressive trimethylation (me3) of lysine 9 (K9) on histone H3 (H3K9me3) in chick embryos [13,34]. This methylation occurs near the transcription start site of crucial neural crest transcription factors, *Sox10* and *Snail2*. The demethylase KDM4A, which removes the trimethylation H3K9me3, enables the expression of *Sox10* and *Snail2* [35]. When the last two are expressed, their gene bodies are subjected to methylation (H3 lysine 36 (K36) me3 or H3K36me3 and H3K9me3, respectively) [35]. *Nsd3* showed increased expression in the neural plate before the initiation of neural crest specifier gene expression, while earlier, *Nsd3* mRNA was most prominent at the borders of the rostral neural plate. Later, its expression was heightened at the dorsal neural tube. As *Nsd1* and *Nsd2* were generally minimally expressed throughout the whole embryo, this points to the fact that *Nsd3* is the main NSD methyltransferase in the neural crest. *Nsd3* actively and directly regulates the expression of *Sox10*, and it secondarily alters the levels of *Snail2*, *Sox9*, or *FoxD3* expression, but it does this in an indirect manner. Consequently, *Nsd3* is crucial and necessary for *Sox10* induction and NC migration [4]. Post-EMT genes are depicted in Figure 1.

## 3. NCPs

### 3.1. Cranial NCPs

#### 3.1.1. Goldenhar Syndrome

Goldenhar syndrome, also known as hemifacial microsomia or oculo-auriculo-vertebral spectrum (OAVS), is a developmental disorder that in the majority of cases is unilateral. It has an impact on the first and the second pharyngeal arch, formed during the fourth week of gestation, possibly because of a deviation from normal angiogenesis [36] together with other external factors that influence vessels, such as drugs, medications and hormones [36,37,38]. Any other deviation from the normal differentiation and ventrolateral migration of the cranial NCCs in order to form the PAs is correlated with OAVS [39,40]. Its phenotype includes implications in the eyes and ears, craniofacial malformations, and various abnormalities in the cardiac, respiratory, gastrointestinal and central nervous system [41,42]. The etiopathogenesis of OAVS is based on a combination of genetic and environmental/developmental factors, although the exact mechanism has not been completely understood yet, with its multisystemic implications remaining without a known origin. As far as the genetic factors are concerned, the *MSX* gene family has been found to be partially responsible for this syndrome [42]. This gene family takes part both at the specification of neural crest borders and in craniofacial development. In frog embryos, *msx1* is needed for the induction of other neural crest border genes including *slug*, *snail* and *foxd3* [10], while in mice, *Msx1* and *Msx2* have active roles in the formation of craniofacial, limb, and neural tube features. Knockout mice for *Msx1* display mandibular and maxillary underdevelopment, with the frontonasal region and the forebrain being smaller than the usual. Exencephaly was present in some mice, along with variations in the size and shape of optic cup, when the neural tube appeared with defects [43]. In humans, *MSX1* mutations are responsible for a phenotype including total dental agenesis and cleft lip and palate [44]. Other characteristics that arise from the abnormal development of the first and second PAs are small ear and body size, a coloboma in the upper eyelid, and an epibulbar dermoid [45]. In some cases, other genes have been found to contribute to OAVS, such as *MYT1* and *SF3B2* [41,46]. Apart from the genetic factors and the process of angiogenesis that were mentioned before, external stimuli that contribute to the OAVS phenotype include mothers diagnosed with diabetes mellitus [38], celiac disease, and hypothyroidism [47].

#### 3.1.2. Axenfeld–Rieger Syndrome (ARS)

ARS is a developmental autosomal dominant disorder [48] of the anterior segment, which mainly affects the craniofacial region and incorporates ocular malformation with posterior embryotoxon and underdevelopment of the iris, polycoria, which can be combined with corectopia. Abnormalities in other systems like the cardiac, dental, and myoskeletal systems can be observed. The pathophysiology of this syndrome is associated with disrupted neural crest migration in the early stages of embryogenesis. Two major genes that have been proved to be responsible for this syndrome are *PITX2* and *FOXC1* [49,50]. Both *Pitx2* and *Foxc1* in mouse embryos are transcription factors that are regulated by *Tgfβ*, a signaling molecule that aids the neural crest cells in their migration, guiding them towards the periocular mesenchyme, and controls ocular development [51]. Further data confirm the involvement of TGFβ (through a pathway that includes *PITX2* and *FOXC1*) in the propriate formation of ocular structures in humans too, thus proving the contribution of these molecules to ocular abnormalities present in ARS [52]. *FOXC1* is also active in premigratory or migrating cardiac neural crest cells as well as in tissues located near the pathways of migration, explaining in this way the nonocular multisystemic impacts of ARS [50]. Other genes that are responsible for some of the aspects of the syndrome in a small amount of individuals include *CYP1B1*, *PRDM5*, *JAG1*, *USP9X*, *CDK13*, *HCCS*, *AMELX* and *BCOR* [50,53].

#### 3.1.3. Craniosynostosis

Syndromic craniosynostosis is an autosomal dominant condition characterized by the premature closure of one or more cranial sutures. Its pathogenesis has its roots in the development of the cranial vault during embryogenesis, and it can be divided into six different subtypes, all of which affect the coronary sutures (Saethre–Chotzen syndrome also affects the frontal suture). All of them are the result of mutations in gene families that affect the EMT process and migration of NCCs. In Pfeiffer syndrome, the skull has a cloverleaf shape, accompanied with intense hypoplasia of the midface and proptosis. The genes that are involved in this syndrome are *FGFR1* and *FGFR2*. Generally, in the pre-EMT stage of embryonic development, *FGF/FGFR* interaction is crucial for the induction of the neural crest and the activation of proneural genes in the neural ectoderm [3,54]. Especially, the *FGFR2* receptor is present in large amounts in the cartilage of the cranial base and is responsible for the proper induction, terminal differentiation, and apoptosis of the precursors of osteocytes, meaning that the early differentiation of cells that express those receptors can cause the early closure of cranial sutures. This syndrome is also associated with other implications including intellectual disability and conductive loss of hearing. Other subtypes of craniosynostosis are affected by *FGFR* genes in a similar way. Namely, Apert syndrome is caused by a mutation in *FGFR2* and includes phenotypic characteristics like cleft palate, hearing loss, and hypertelorism, while Crouzon syndrome is a result of *FGFR2* and *FGFR3* mutation, presenting with mandibular prognathism, tarsal bones’ fusion, and hearing loss. Moreover, Muenke syndrome is a subtype of craniosynostosis induced by *FGFR3* mutation and characterized additionally by macrocephaly, carpal and tarsal bones’ fusion, and hypertelorism [55]. Another gene of embryonic origin that participates in this family of craniofacial abnormalities is *TWIST*. Apart from its role in the pre-EMT phase of neural crest development, when it cooperates with *Sip1/Zeb2*, *Sox9/10* and *Snai1/2* in order to decrease the expression of Cadherin-6B and other epithelial cadherins and upregulate the transcription of more mesenchymal cadherins in vertebrate embryos [56], in humans, it is a negative regulator of the *FGF/FGFR* expression, thus determining the extent and the time of osteocytic differentiation. Mutations in *TWIST1* are indicative of Saethre–Chotzen syndrome, having an phenotype that is analogous to Muenke syndrome in addition to small ear size, a lower hairline, and syndactyly [55]. Craniofrontonasal syndrome is the last category of craniosynostosis, and it is characteristic of mutations in *EFNB1*. The normal allele takes part in the process of bone formation. Specifically, *EFNB1* is expressed by osteoblast progenitors that arise from NCCs and is responsible for increasing the thickness of the bones through upregulating the transcription of *SP7*, a key gene in bone formation. In avian embryos, the distinction between neural and non-neural crest cells and the kickstart of migration is controlled by ephrin-B1 and ephrin-B2. Particularly, crest cells that migrate express ephrin-B2, while, on the other hand, the formation of borders that indicate the migration pathways consist of non-neural crest cells that express ephrin-B1 [57]. In humans, the equivalent receptor ligand, *EFNB1*, participates in neural crest adhesion and migration routes as well as in normal bone formation. Its mutations are responsible for the disruption of proper osteogenesis, leading to the disrupted development of long bones, of osteoblast/osteoclast balance, and of the growth plate [58]. Thus, such mutations in *EFNB1* can be linked to CFNS, characterized by a wide nasal bridge, brachycephaly, a nasal tip that is wide or divided, and even consequences for the bones and joints [55].

#### 3.1.4. Craniofacial–Deafness–Hand Syndrome (CFDS)

CFDS is a rare disorder mainly affecting the craniofacial region of the developing embryo, with traits like intense hearing defects, malformed facial structures including hyperteloroism, small mouth size, missing or underdeveloped wrist and nasal bones, and hand abnormalities [53,59]. The gene responsible for this syndrome is *PAX3*, which plays a major role at the pre-EMT phase in many species. In Xenopus, *pax3* in accordance with *zic1* kickstarts the differentiation of the neural crest cells [3]. Mutation in *PAX3* in humans can lead to various facial dysmorphisms along with symptoms from other systems, thereby causing CFDS [60].

#### 3.1.5. Tricho-Dento-Osseous Syndrome (TDO)

TDO is an uncommon, penetrative, autosomal dominant disorder, with its basic phenotype enclosing abnormal formation of the hair, dental structures (taurodontism, enamel hypoplasia) and skull, featuring augmented bone density [61,62]. The gene that is involved in this syndrome is *DLX3*, a member of the *DLX* family that is vital for the determination of the dorsal–ventral axis and for the formation of the structures coming from Pas during embryogenesis. In mouse embryos, the domains where *Dlx3*, together with *Dlx4*, is active, are restricted to the farthest tip of the lower jaw structure, inhabiting the ventral region of the PAs. Their activity and their contribution to the formation of the lower jaw structure are stimulated or sustained by other members of the *Dxl* family, including *Dlx5* and *Dlx6*, which are located in the first PA [63]. Findings regarding the contribution of *DLX3* to human neural crest development and PA formation are similar to those obtained in rodent models. *DLX3* starts its expression early in the first and second pharyngeal arches, from which odontoblasts and other craniofacial traits come. Its induction in later stages is crucial for the epithelium, placodes, limbs, hair, and the earliest developmental stage of teeth, contributing also to the formation of enamel [64]. Multiple mutations of this gene lead to abnormal odontoblast differentiation and enamel formation, taurodontism, and wavy hair immediately after birth [65]. These traits are necessary for the diagnosis of TDO, whose first clinical manifestations are usually found post birth and during the first year of life [66].

#### 3.1.6. Peter’s Anomaly

Peter’s anomaly features a dysmorphic central cornea and malformed anterior eye segment that includes the corneal posterior stroma, endothelium, and Descemet’s membrane [53]. Peter’s anomaly can be divided into three subcategories: Peter’s anomaly type I, whose phenotype includes central corneal opacity with iridocorneal adhesions; Peter’s anomaly type II, which features central corneal opacity accompanied by cataracts or corneolenticular adhesions; and Peter’s-plus syndrome, which combines the syndrome with facial abnormalities such as cleft lip/palate and abnormal ear morphogenesis, heart defects, and intellectual disability and developmental delay [67]. The homeobox genes *PAX6*, *PITX2*, *FOXE3*, and *FOXC1* are responsible for the emergence of the syndrome, as the dysmorphogenesis of the lens vesicle and its failure to separate from the surface ectoderm is attributed to their mutations [51,52,68]. *PAX6* is one of the genes necessary to begin human eye formation and lens differentiation [39], and its homologous gene *eyeless* in Drosophila can alone induce eye morphogenesis [69]. Moreover, it has been observed in zebrafish embryos that two distinct neural crest streams enter the optic cup, with *pax6* being necessary for their proper guidance. As *pax6* is responsible for the appropriate guidance of these two neural crest streams, as well as for the expression of guidance molecules that aid this procedure, its loss can lead to serious anterior segment abnormalities [39]. Thus, *PAX6* mutations can impede eye formation and contribute to the malformed anterior eye segment present in Peter’s anomaly. Another one of the genes connected to Peter’s anomaly, *FoxE3*, is placed downstream of *Pax6* in mice studies [40]. In humans, *FOXE3* is important for eye morphogenesis, with its expression being confined mainly to the lens, particularly in the anterior lens epithelium, albeit with some presence observed in the posterior region. Mutations in FOXE3 can result in various congenital eye abnormalities, leading to the phenotype of Peter’s anomaly [40]. *Pitx2* and *Foxc1* in mouse embryos are transcription factors and are both regulated by Tgfβ, a signaling molecule that aids the NCCs in their migration, guiding them towards the periocular mesenchyme and that controls ocular development, a fact that explains their involvement in ocular anomalies [51]. Certain mutations of *CYP1B1* are also connected to the syndrome [70], while *B3GLCT* mutations are connected to Peter’s-plus syndrome, establishing it as a glycosylation disorder [71].

#### 3.1.7. Bamforth–Lazarus Syndrome

The observable characteristics of this condition include abnormalities such as thyroid malformation, with resulting hypothyroidism; split palate; micrognathia; and coarse hair texture, sometimes accompanied by either choanal atresia or a divided epiglottis [72]. Patients with BLS exhibit various mutations in the *FOXE1* gene, which produces a forkhead transcription factor [53]. In mice, *Foxe1* is found in the endoderm of the foregut and in the ectoderm of the craniopharyngeal region crucial for palate development, and it is present in the early stages of thyroid formation. Human *FOXE1* expression was observed in the epithelial lining of the oropharynx and in the thymus, but its onset was delayed compared to mice [73]. Hair defects in BLS patients are most likely connected to *FOXE1*, as it is involved in hair follicle morphogenesis downstream of the SHH/GLI pathway [74]. Split palate and micrognathia can be attributed to the disruption of facial chondrogenesis, which has been observed in zebrafish. In the latter, when *foxe1* has been knocked down, shortening of the Meckel’s cartilage and inverted or shortened ceratohyal cartilages are exhibited [75]. When it comes to palate formation, *Foxe1*-null mice feature cleft palate and thyroid malformation, the same severe features as patients with the orresponding syndrome [76]. Furthermore, during embryogenesis, *FOXE1* regulates *MSX1* and *TGF-b3*, which take part in cranial development and are expressed in vertebrate embryos at epithelial–mesenchymal points of contact. Consequently, *FOXE1* mutations and their indirect effect on *MSX1* and *TGF-b3* might explain some of the facial deformities in this syndrome. In mice, *Msx1* is important for mandible, maxilla and tooth formation, while *Tgf-b3* is crucial for the morphogenesis of neural crest-derived eye structures [73]. In chicken embryos, *Msx1* is a pre-EMT neural plate border gene, which appears to be influenced by WNT signaling and is necessary for the activation of the *Est1* gene [3]. Moreover, *Msx1* is expressed in the distal incisal mesenchyme in mammals, something that indicates its role in tooth formation and incisors in particular [32]. Lastly, while *Msx1* has not been found to be expressed in cardiac NCCs, it has been found active in NCCs arriving at the heart to create its outflow tract [77].

#### 3.1.8. Branchio-Oculo-Facial Syndrome (BOFS)

BOFS is an autosomal dominant congenital disorder characterized by dysmorphogenesis of the first and second PA [53]. The most prominent characteristics are craniofacial abnormalities, which include supra-auricular sinuses, malformed auricles, external and middle ear anomalies, microphthalmia, lacrimal duct obstruction, abnormal philtrum, and pseudocleft of the upper lip, sometimes accompanied by intellectual disability and developmental delay [78]. This syndrome’s pathogenesis is connected to the *TFAP2A* gene, as mice lacking *Tfap2a* display atypical facial neural crest-derived structures [79]. Its importance in craniofacial development arises from its involvement in cranial closure and the morphogenesis of the facial prominences and the lens vesicle [80,81]. *Tfap2a* is expressed in both pre-migratory and migratory NCCs [82], while it has been found that TFAP2A is also present in migratory human neural crest [83]. In particular, mice lacking *Tfap2a* display atypical facial neural crest-derived structures [79]. In zebrafish, genes of the *tfap2* family are neural plate border specifiers and play a crucial role in the activation of neural plate border specification [3], while zebrafish embryos injected with anti-tfap2 morpholinos display severely dysmorphic Pas, showing that tfap2a expression is vital for the proper development of craniofacial structures [84]. This gene family is also implicated in cardiac development, as gene mutations can cause patent ductus arteriosus [33].

#### 3.1.9. Cerebral Autosomal Dominant Arteriopathy with Subcortical Infarcts and Leukoencephalopathy (CADASIL)

CADASIL is an autosomal dominant small vessel disease that affects the brain. It is an arteriopathy that causes subcortical infarcts, migraines, and restriction in blood supply to cerebral tissues; it may manifest as cognitive decline, psychiatric symptoms, and leukoencephalopathy due to the degeneration of vascular smooth muscle cells and pericytes [53,85]. This degeneration is mostly dependent on *NOTCH3* mutations. While *NOTCH3* is expressed in the nervous system of the embryo, in adult life, it is mainly expressed in vascular smooth muscle cells [86]. Due to *NOTCH3* mutations, the extracellular domain of the Notch3 receptor, which contains EGF-like repeats, is accumulated between the smooth muscle cells of the brain’s blood vessels, leading to their degeneration and, consequently, causing restriction of blood flow [86,87]. Since the receptor protein produced by the *NOTCH3* gene is present not just in VSMCs but also in pericytes, pericytes and the capillary vessels they support can also be affected by CADASIL [88]. The role of *NOTCH3* during embryogenesis is mainly in VSMCs differentiation, with *Notch3* being an important factor in proliferation and the impediment of VSMCs apoptosis [89,90]. *Notch3*-null mutant mice are characterized by loss of VSMCs, blood–brain barrier disruptions, and decreased vessel integrity in the central nervous system, characteristics found in patients with CADASIL [91]. During brain morphogenesis, NOTCH signaling is crucial for mural cell (also known as vSMCs and pericytes) differentiation from neural crest-derived stem cells. It has been shown, using hPSC-derived neural crest, that NOTCH signaling is a deciding factor in driving the terminal differentiation of mural cells in the face and the forebrain [92,93]. *FOXC1* and *PITX2* are two other genes that are implicated in small-vessel disease, with both of them being expressed in NCCs. *FOXC1* it contributes to vascular stability, and its aberrant function contributes to cerebral vascular diseases like CADASIL. It also physically interacts with *PITX2*, which produces a transcription factor and regulates vascular smooth muscle cell proliferation. *PITX2* mutations can lead to phenotypes with cerebral vascular dysfunction, similar to the CADASIL phenotypes [94]. Lastly, TIMP3 and vitronectin are two extracellular proteins that might play a role in CADASIL pathogenesis. Reducing TIMP3 and vitronectin levels in mice can prevent cerebral blood flow deficits and white matter lesions, leading to the conclusion that increased levels of TIMP3 and vitronectin contribute together with NOTCH3 mutations to CADASIL’s arteriopathy [95].

#### 3.1.10. Congenital Aniridia

Congenital aniridia is an uncommon autosomal dominant eye condition characterized by the complete or partial absence of the iris. This disorder affects numerous eye components. Typically, there is underdevelopment of the fovea, which often leads to nystagmus and diminished vision. Aniridia-related complications such as glaucoma, keratopathy, and cataracts are severe and progressive, potentially exacerbating visual impairment. The underlying cause of aniridia is a mutation in the *PAX6* gene, a deletion that leads to a premature stop codon [96]. *PAX6* is a crucial gene in the proper development of the human eye, with experiments in vertebrates confirming its significance. Specifically, in zebrafish embryos, *pax6* is necessary for the normal formation of the anterior segment of the eye. In the eye development, two distinct neural crest streams have been found to enter the optic cup; the first reaches the proximal region and is restricted there, while the second population migrates in the distal anterior segment. *pax6* is responsible for the appropriate guidance of these two neural crest streams and for the expression of guidance molecules that aid this procedure, with its loss being the cause for serious anterior segment abnormalities leading to the congenital aniridia phenotype with underdeveloped fovea, corectopia, reduced visual sharpness, and nystagmus [97,98]. In humans, the phenotype of aniridia has also been linked to mutations in other genes like *FOXC1*, *PITX2*, *FOXD3*, *TRIM44*, *ELP4*, *DCDC1*, *CYP1B1* [96,99,100]. *PITX2* takes part in the proper development of structures of the anterior segment of the eye in embryogenesis as well, with its mutations being responsible for abnormalities in the development of the eyes, teeth, and umbilicus; at the same time, mutations in *FOXC1* result in eye, heart, and hearing defects.

#### 3.1.11. Frontonasal Dysplasia (FND)

Frontonasal dysplasia consists of a broad spectrum of craniofacial anomalies. It comprises various defects of the craniofacial midline, and its phenotype includes hypertelorism, a broad nasal root, a lack of nasal tip, facial clefts, anterior cranium bifidum, and widow’s peak [101,102]. There are three types of FND. FND1, which is characterized by a distinct concave shape of the nasal tip, is attributed to *ALX3* [103]. FND2, which is characterized by craniosynostosis, cranium bifidum, and brain abnormalities, is a result of *ALX4* mutations [104]. FND3, which is the most severe phenotype of frontonasal dysplasia, is caused by loss of the *ALX1* gene, which results in complete failure of fusion of the frontonasal process and the maxillary arch. The *Alx* genes produce homeodomain-containing transcription factors and are expressed in broad overlapping domains in cranial NCCs. In mouse embryos, *Alx1*, *Alx2*, and *Alx3* are expressed in NCCs in the frontonasal process [105], something that highlights their connection to frontonasal dysplasia, while in zebrafish, *alx1* is present in migrating NCCs during the first stages of migration [106]. During craniofacial development, *Alx3/4* are present in the first PA and contribute to the formation of the lower jaw under the regulation of *Dlx5/6* [107]. Independently of *Alx3*, *Alx4* is also expressed in the distal incisor mesenchyme and contributes to the development of incisor morphology [63]. Therefore, the *Alx* gene family is closely related to the morphogenesis of neural crest-derived tissues, and *Alx* mutations can lead to defects in the craniofacial midline, facial clefts, and lack of nasal tip. Additionally, mutations in *Shh*, *Bmp4*, and *Fgf8* can be connected to FND [101]. The coordinated expression of genes such as *Shh*, *Bmp4*, and *Fgf8* is pivotal in establishing the morphogenesis of the facial primordia. Disruption of this coordination can lead to frontonasal process defects and facial clefting, like in FND. Specifically, the expression domains of *Fgf8* and *Shh* in the frontonasal ectoderm form a signaling center called the frontonasal ectodermal zone, which guides neural crest’s migration [108,109]. Moreover, *Fgf8* and *Bmp4* are crucial for the patterning of the mandible mesenchyme, whose formation and proliferation are disrupted in FND. Proximal mandible mesenchymal markers are stimulated by *Fgf8*, which is secreted by the proximal epithelium, while distal mesenchymal markers are induced by *Bmp4*, secreted by the distal epithelium [63]. Disrupting the complicated expression of Fgf8 and Bmp4 in chicken embryos can lead to lack of development in the midface, like in patients with FND that feature hypertelorism, a broad nasal root, and clefting [110]. *EFNB1*, which is expressed in cells surrounding the streams of cranial NCCs, might also be related to the pathogenesis of this dysplasia, as female patients with *EFNB1* mutations exhibit a more severe phenotype [111]. Another relative gene is *Kif3a*, with mice lacking neural crest *Kif3a* expression featuring a wider frontonasal process and facial clefts, similar to the phenotype of human frontonasal dysplasia [112].

#### 3.1.12. Hajdu–Cheney Syndrome

Hajdu–Cheney is a rare autosomal dominant disorder accompanied by arthrodentoosteodysplasia, which causes specific craniofacial defects, cardiovascular defects, osteoporosis, and kidney cysts [53]. Hajdu–Cheney is characterized by increased bone remodeling and decreased bone formation, something that explains skeletal defects related to low bone density. *NOTCH2* gain-of-function mutations are linked to this syndrome [113]. *NOTCH2* is generally implicated in cardiac neural crest development [114,115], as NOTCH signaling takes part in the differentiation of cardiac NCCs and outflow development. Disruption of NOTCH signaling is linked to cardiac dysmorphogenesis and therefore could be related to the cardiovascular defects in Hadju–Cheney [53]. After the migration of the cardiac NCCs, NOTCH receptors and ligands are expressed in the outflow tract and the developing arteries [116] and play a crucial role in the differentiation of smooth muscle cells [117]. NOTCH signaling partakes in skeletal morphogenesis, chondrogenesis and in osteoblast and osteoclast differentiation [118,119,120], which could impact craniofacial formation, leading to Hajdu–Cheney. Blocking NOTCH signaling or *NOTCH2* leads to problematic enamel formation, while it also causes the death of dental epithelial stem cells in mouse incisors [121,122], leading to the dentodysplasia present in Hajdu–Cheney patients.

#### 3.1.13. Moebius Syndrome (MBS)

MBS is a non-progressive neurological condition that stems from the underdevelopment of facial nerves and is accompanied by strabismus, hypertelorism, and partial facial paresis, which presents itself as mask-like facies and strained control of eye movements [123]. Mostly affected are the trigeminal, abducens, and facial nerves. Some patients also feature microstomia, cleft palate, lingual dysplasia, and epicanthic folds [124,125]. *MBS1*, *MBS2*, and *MBS3* are the genetic loci most prominently linked to the syndrome, but the specific genes and their connection to the neural crest remains to be elucidated [126,127]. Moreover, mutations of another gene, *TUBB3*, which is expressed in premigratory NCCs and later present in neural crest-derived neurons [21], are linked to MBS [128]. A mouse study has connected this phenotype with elevated SHH signaling in combination with correlated decreased WNT signaling [129]. *SHH* levels are essential for proper neural crest cell differentiation, particularly in the development of the head and face. When *Shh* is absent in mutant mice and humans (*SHH*), it leads to conditions like holoprosencephaly and cyclopia, where the hemispheres fail to separate correctly [130]. Enhanced SHH signaling affects the proliferation and migration of NCCs and impedes the interactions between NCCs and the placodes and is correlated with disorganized trigeminal and facial nerves, which are affected in MBS [129]. In cranial morphogenesis, WNT signaling is important for the delamination of NCCs and later determines the sensory fate of neural progenitors [51]. Disruption, therefore, of WNT signaling renders nerve abnormalities like those in MBS probable. The implication of *HOX* family genes, which are key genes in the morphogenesis of the cranial neural crest, is also likely, with emphasis on *HOXA1* and *HOXB1* [127,131,132]. Mutations in *PLXND1* and *REV3L* account for a percentage of MBS patients. *Plxnd1*-null mice exhibit hypoplasia of the neural fibers that connect areas of the brain as well as disruption of neuronal migration; meanwhile, in *Rev3l*-mutant mice, brain volume is decreased. Therefore, the clinical features of MBS patients are similar to those in mutant mice models [106]. Lastly, Moebius syndrome is connected to the genes *GSH1*, *CDX2*, *CRBP1*, *PBX2*, *EGR2*, and *SOX14* [133,134,135].

#### 3.1.14. Pierre Robin Sequence (PRS)

PRS is a rare congenital condition, including the clinical triad of micrognathia, cleft palate, and glossoptosis, which carries the risk of mechanical airway obstruction. It can be divided into two subtypes; the isolated, nonsyndromic and the syndromic, usually accompanied by genetic syndromes like Stickler syndrome, velocardiofacial syndrome, and Treacher Collins syndrome [136]. Among the cases that are reported, the genetic factor that is responsible for PRS seems to be a mutation in the *SOX9* gene, which is important during embryogenesis for the formation of chondrocytes deriving from multipotent NCCs [137]. In mouse embryos, *Sox9* is expressed in cranial NCCs, which are responsible for the proper formation of the cranial and facial skeleton [3]. Contribution of *SOX9* in the differentiation of cranial NCCs into chondrocytes and in the formation of many craniofacial structures could explain the micrognathia and glossoptosis observed in PRS. An additional role of *Sox9* is to positively regulate the expression of *Col2a1*, *Col11a1*, and *Col11a2* in both mice and chicken (*SOX9 COL2A1*, *COL11A1*, and *COL11A2*, respectively) embryos in the process of chondrogenesis, with their mutations leading to PRS as well [138,139]. Another genetic locus found to play a significant role in PRS is *BMP2*. It is a member of the BMPs family, which is necessary for the proper formation of the palate. Specifically, in mouse embryos, *Bmp2* is present in NCCs that give rise to chondroblasts and osteoblasts, giving them the ability to proliferate and differentiate into their terminal fate. As a consequence, in mouse mutants, a lower rate of cell proliferation results in a decrease in osteogenic and chondrogenic precursors arising from NCCs, contributing to the development of a smaller mandible. This, in turn, hinders the descent of the tongue and ultimately leads to the formation of a cleft palate. Additional mutated genes in mice that are considered to contribute to the cleft palate phenotype are *Prdm16* and *Tak1*, found in the TGFβ signaling pathway [140,141], as well as *Erk*, a member of the BMP, TGFβ, FGF, and EGF pathways [142].

#### 3.1.15. Mowat–Wilson Syndrome

Mowat–Wilson syndrome is a disorder characterized by abnormal facial traits including eye hypertelorism, prominent chin, a mouth-open expression, earlobes located higher than usual, and underdevelopment of the optic nerve [143,144]. It is also accompanied by congenital hearing loss, cardiovascular and genitourinary abnormalities, HSCR, and impaired intellectual sharpness [143]. It is caused by mutations in the gene *ZEB2* [145,146], which is expressed in different domains during embryogenesis. In mice, it is important for the proper development of the neuroepithelium [147] the neural plate, and as a result the NCCs, cortex of the brain, and the mesoderm [148]. In humans, the *ZEB2* gene plays a central role in coding for the Smad interaction protein 1 (SIP1), which is crucial for the appropriate formation of different components within the eye during development. In particular, SIP1 activates the TGF-β pathway and promotes the proliferation and differentiation of NCCs, assuring normal organogenesis. The importance of *ZEB2* in the formation of the craniofacial domain, part of the nervous system and the mesenchyme, leads to the phenotype of this syndrome, with eye abnormalities affecting the formation of the lens, causing congenital cataract. Increased expression of these molecules in the eyeball, NCCs, genital glands, and musculoskeletal system comes in accordance with the eventual harm inflicted upon these cells, directly aligning with the range of abnormalities observed in MWS [144].

#### 3.1.16. SAMS Disorder

SAMS disorder is an autosomal recessive syndrome that causes short stature, auditory canal atresia, mandibular hypoplasia, and skeletal abnormalities, including femoral, humeral and pelvic abnormalities [149]. Mutations in *GSC*, which encodes Goosecoid homeodomain transcription factor, are responsible for this phenotype [150]. *gsc* is required for mesodermal organization during Xenopus gastrulation [125], while later in mouse embryos, its expression is detected in the first pharyngeal arch, the mandible, the auditory canal, and the limb buds [151]. *Goosecoid* is involved in the patterning of the pharyngeal arches, thus placing SAMS in the category of NCPs [149]. This pattern of expression of *GSC* is in accordance with the mandibular, auditory and limb anomalies found in patients with SAMS disorder. *Gsc*-knockout mice exhibit craniofacial anomalies, limb defects and skeletal malformations in humeri and femora, also compatible with the phenotype of patients with SAMS disorder [152,153]. *Gsc* is also connected to EDNRA signaling, as its expression is absent after loss of EDNRA signaling [154], while in embryos where both *Ednra1* and *Ednra2* are depleted, there is a notable decrease in *Gsc* expression, specifically within the ventral regions of the mandibular and hyoid arches [155]. When *Gsc* is not expressed in ET-/- mice embryos, craniofacial abnormalities arise, connecting *Gsc* with SAMS disorder [154]. Therefore, apart from *GSC*, EDNRA signaling might also play a part in SAMS pathogenesis.

### 3.2. Cardiac (Vagal) NCPs

#### 3.2.1. DiGeorge Syndrome

**Figure 2:**
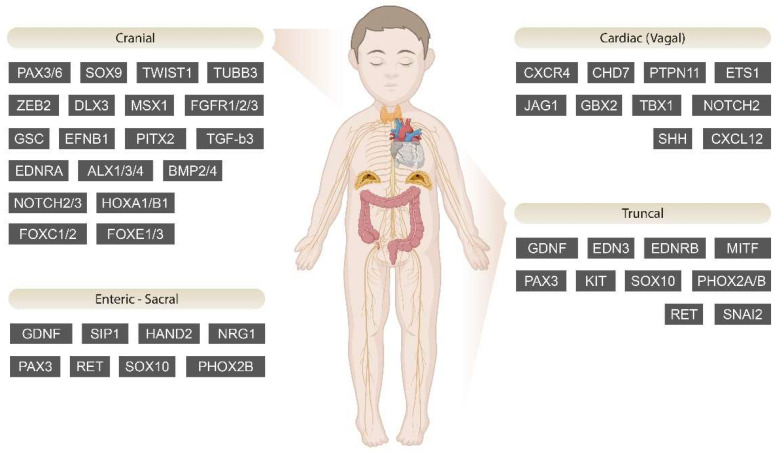
Region-based genes implicated in the differentiation of NCCs during the late phase of neural crest-derived tissues formation. Cardiac (vagal) genes refer to the formation of the heart and outflow tract, whereas Enteric–sacral genes contribute to the development of ganglion cells in the myenteric and submucosal wall plexuses/layers of the lower gastrointestinal tract.

Several cardiocraniofacial defects are present in this syndrome, such as truncus arteriosus, tetralogy of Fallot, and double-outlet right ventricle; hypothyroidism, hypoparathyroidism, and a hypoplastic thymus are also present [117]. *TBX1* is a gene whose product is T-Box transcription factor; it has a significant role in cardiac neural crest migration and final differentiation, connected to the pathophysiology of this syndrome [77]. During migration, while it is not significantly expressed by cardiac NCCs themselves, it is vastly present in the surrounding environment and in particular the pharyngeal ectoderm, endoderm and the second heart field; thus, it guides the cardiac NCCs to their final destination [117]. When *TBX1* expression is decreased, due to mutations or deactivation, migration of cardiac NCCs is not concluded and their maturation is not finalized due to absent communication between the neural crest and the mesenchyme, which leads to decreased activation of the MAPK pathway and early expression of the BMP pathway [77]. In mutant *Tbx1* mice, altered neural crest migration has been observed in the third, fourth, and sixth PAs [156]. Additionally, *Tbx1* deletion has been proven to result in aorticopulmonary defects and incomplete morphogenesis of the aortic arch and the major arteries, due to decreased cardiac NCC migration in the cardiac field [77]. The indirect action of *Tbx1* is manifested through its effect on the expression of *Gbx2* in the pharyngeal ectoderm, which most likely interferes with the migration of cardiac NCCs by potentially reducing the activity of the SLIT/ROBO signaling pathway and perhaps downregulating the Slit2 ligand [157]. Apart from *Gbx2*, *Tbx1* also affects *Cxcr4* and *Cxcl12*, which encode the receptor for the chemokine CXCL12/Stromal cell-derived factor 1 and the CXCL12 chemokine, respectively [158]. In zebrafish, *cxcr4* is present in the NCCs heading towards the PAs, while its receptor cxcl12 lies in the endoderm of the PA [107]. At the same time, the chemotaxis orchestrated by Cxcr4 plays a crucial role in the formation of neural crest-derived structures in the placodes [25]. These genes in mice appear to be downstream of *Tbx1*, and their inactivity results in disrupted neural crest migration in the PAs and major cardiocraniofacial abnormalities [158]. This complex web of interactions explains to some extent the cranial and cardiac defects found in patients with DiGeorge. Disease-related genes implicated in NCCs’ differentiation are depicted in Figure 2.

#### 3.2.2. CHARGE Syndrome

The phenotype of this syndrome, from which its name derives, includes coloboma, heart anomaly, atresia of choanae, intellectual and physical disability, genital hypoplasia, and ear anomalies (with cleft palates also being mentioned) [159]. This syndrome’s cardiac anomalies and outflow tract defects, which stem from the cardiac NCCs genes *TBX1* and *CHD7*, are similar to the ones present in DiGeorge and velocardiofacial syndrome. However, the broader spectrum of organs it encompasses means an earlier impact on development or a bigger expression pattern of the genes involved [117]. *CHD7*, which belongs to the chromodomain helicase-binding family [22], is expressed in Xenopus and mice in premigratory and migratory NCCs [23], is present in neural crest-derived tissues in mice [24], and is involved in melanocyte differentiation in zebrafish [160]. Xenopus *chd7* in particular is crucial for the activation of several post-EMT genes, including *sox9*, *twist* and *snail2*. In humans, the morphogenesis of the trachea and the craniofacial cartilage is severely dependent on *CHD7* [53]. Mice with a heterozygous mutation in *Chd7* exhibit inconsistent dysmorphogenesis in tissues typically impacted in CHARGE syndrome, such as the eye, inner ear, heart, craniofacial tissues, and genitalia. These manifestations include cardiovascular abnormalities, like heart septal defects and hemorrhages, issues like choanal atresia and cleft palate, and genital abnormalities [161].

#### 3.2.3. Velocardiofacial Syndrome

This syndrome shares a lot of similarities with DiGeorge syndrome, both in phenotype and in the embryological genes that influence its pathogenesis. The same cardiac anomalies—truncus arteriosus, tetralogy of Fallot, and double-outlet right ventricle—are present, but velocardiofacial syndrome is also characterized by facial anomalies such as cleft lip and cleft palate [117]. *TBX1* is the gene most associated with this syndrome, as it has been proved that its deletion results in aorticopulmonary defects and incomplete morphogenesis of the aortic arch and the major arteries due to decreased cardiac neural crest presence in the cardiac field [22,77].

#### 3.2.4. Fetal Alcohol Spectrum Disorders

Fetal alcohol syndrome, the most extreme phenotype of fetal alcohol spectrum disorders, presents with cardiac anomalies [117]; dysmorphic facial features such as flat philtrum, low nasal bridge, flattened lip, thin maxilla, and cleft palate; skeletal anomalies; ear malformations [162]; growth deficiency; microcephaly; and developmental delays [117]. The involvement of the neural crest is prevalent, as several of these characteristics arise from reduced cranial and cardial neural crest migration [53]. The pathophysiology of this syndrome derives from the effects of ethanol on the neural crest. Ethanol causes apoptosis in cranial NCCs as it binds to the L1 adhesion molecule present on them [22]. Ethanol also disrupts morphogenesis by inhibiting midline expansion and SHH signaling. Ethanol disrupts the SHH protein while at the same time suppressing *SHH* expression by activating protein kinase A, which represses *SHH* [163]. In mouse embryos, it has been concluded that SHH signaling, combined with its effect on BMP signaling and *Sox9* expression, is vital for PA formation and cardiogenesis. Disruptions in this pathway lead to this specific phenotype, which consists of cardiac anomalies, maxilla malformations, and cleft palate. When *Shh* is ablated, loss of NCCs is observed, and cardiac defects similar to tetralogy of Fallot are present [164]. In mice, *Shh* is expressed in large quantities in PA1, while the *Shh* signaling cascade also includes *Tbx1*, which acts downstream of *Shh* [165]. This involvement of *Tbx1* may possibly explain the aorticopulmonary defects and the malformations of the aortic arch that are sometimes present. *Shh* is also implicated in the development of the enteric nervous system, as it participates in the activation of the *Foxf1* and *Foxf2* forkhead transcription factors [166], something that might later explain other characteristics of this phenotype, such as chronic intestinal pseudo-obstruction [167].

#### 3.2.5. Alagille Syndrome

Alagille syndrome, also known as arteriohepatic dysplasia, manifests as cardiac outflow tract defects, aortic valve hyperplasticity, tetralogy of Fallot, peripheral pulmonary artery stenosis, pigmentary retinopathy, posterior embryotoxon, and dysplastic kidneys. There are two common mutations that cause this syndrome: mutations in *JAG1*, which cause Alagille syndrome Type 1, and mutations in *NOTCH2*, which cause Alagille syndrome Type 2 [117]. NOTCH signaling has been implicated in the differentiation of cardiac NCCs and outflow development; thus, its impairment is connected with cardiac dysmorphogenesis [53], but it has not been connected to cardiac NCCs’ migration [22]. After the migration of the cardiac NCCs, NOTCH receptors and ligands are expressed in the outflow tract and the developing arteries [116] and play a crucial role in the differentiation of smooth muscle cells [117]. *JAG1* is the most present mediator of NOTCH signaling [168]. Mutations in *JAG1* cause blood vessel formation abnormalities, including aortic valve coarctation, that contribute to the basic phenotype [169].

#### 3.2.6. Noonan–LEOPARD Syndrome

Noonan syndrome is a genetic disorder characterized by cardiac defects, postnatal developmental delay, renal malformations [170], and facial and skeletal abnormalities [117]. These cardiac defects include pulmonary stenosis and septal defects [22], combined sometimes with aortic coarctation and hypertrophic cardiomyopathy [117]. These cardiac anomalies are also present in patients with LEOPARD syndrome, which is a similar condition whose acronym stands for lentigines, ECG conduction abnormalities, ocular hypertelorism, pulmoic stenosis, abnormal genitalia, retardation of growth, and sensorineural deafness. The gene most commonly mutated in these two syndromes is *PTPN11* and its protein (namely SHP2), which encodes a tyrosine-phosphatase with a role in the RAS-MAPK (ERK) pathway [171]. Noonan syndrome is typically associated with increased signaling through this pathway, while mutations in LEOPARD typically result in an inactive SHP2 protein. It has been shown in mice that *PTPN11* and SHP2 are important, not only for the completion of the role of cardiac neural crest cells and the morphogenesis of the cardiac outflow tract [117] but also for the ossification of neural crest-derived cranial bones. Thus, mutations in this gene lead to the aforementioned facial abnormalities [172]. The RAS-MAPK (ERK) pathway and its increased or decreased activity is the catalyst for these two syndromes. Decreased MAPK signaling results in diminished NCC presence in the heart, while the maturation and specification of cardiac NCCs is obstructed, presenting great vessel malformations and valve defects as a result [77]. Moreover, disruption of the ERK/MAPK signaling pathway in the embryonic stages hinders neural crest development, resulting in abnormalities in cardiac, craniofacial, and central nervous system structures and leading to a phenotype compatible with Noonan syndrome. Other pathogenic genes that are intertwined with this disease and affect RAS-MAPK signaling are *SOS1*, *KRAS*, *NRAS*, *RAF1*, *BRAF*, *SHOC2*, *MAP2K1*, and *CBL* [53].

#### 3.2.7. Jacobsen Syndrome

The most common features of this syndrome include cardiac malformations; facial deformities; kidney, genitalia and gastrointestinal tract abnormalities; and hematological conditions, which are present at birth [173]. Loss of *ETS1* is largely responsible for at least a percentage of congenital heart defects as it is normally expressed in the cardiac neural crest, while this gene also plays a role in the coexisting immunodeficiency in Jacobsen syndrome [174]. During embryogenesis, *Ets1* is expressed in chick NCCs before EMT as they move further away from the neural plate border [3]. At a later stage, *Ets1* is responsible for the final stage of migration of cardiac crest cells and the determination of their fate; in its absence, crest cells cannot reach their final destination, which in this case is the conal cushions. In knockout mice, *Ets1* deletion resulted in a phenotype with double-outlet right ventricle, confirming the involvement of *Ets-1* in cardiac abnormalities [174]. Furthermore, during the initial stages of mouse heart development, *Ets-1* is active in both the endocardium and cardiac neural crest-derived structures. When *Ets-1* is deleted in mice, it frequently leads to significant membranous ventricular septal defects and a split cardiac apex, while occasionally resulting in a left ventricle that fails to form an apex. The same mechanism, therefore, could be applicable in human cardiac dysmorphogenesis [175]. Moreover, *Ets1* is regulated by the MEK/ERK signaling pathway in order to prevent NCCs from forming cartilage [168].

### 3.3. Truncal NCPs

#### 3.3.1. Waardenburg Syndrome (WS)

WS is an autosomal dominant disorder accompanied by a phenotype that includes sensorineural loss of hearing and atypical pigmentation of the iris of the eye, of the hair, and of the skin. Its diagnosis is based on major and minor criteria. The major include different iris color, hair pigmentation defects, sensorineural deafness, relocation of the inner canthus, and a diagnosed first-degree relative. The minor criteria involve differentiations in the width of the nasal root, synophrys, early acquisition of gray hair, and underdevelopment of nasal alae. This syndrome is divided into four subtypes depending on the phenotype and clinical signs. WS1 requires two major criteria for its diagnosis (or, alternatively, one major together with two minors). It is characterized by a wide nasal root, dystopia of the canthus, and brief retropositional maxilla and philtrum. WS2 involves differences in iris color and sensorineural loss of hearing, while WS type 3 is similar to WS1 but is correlated with abnormalities in the myoskeletal system. WS4 has traits like WS2, with the distinction based on the presence of HSCR, which is present only in WS4 [176,177]. The gene that is responsible for WS1 is *PAX3*, which, apart from its functions reported in the CFDS, plays a crucial role in the pigmentation process during embryogenesis too. At the time of melanocytic differentiation in mice embryos, *Pax3* increases the survival rate of melanocyte precursors that are formed from NCCs and activates the *Mitf* promoter, another important gene in the pigmentation procedure, in cooperation with *Sox10.* Mutations in either of the genes that are involved in this process cause white coat color phenotypes in mice [178], thus accounting for the pigmentation defects in humans with mutations in the *PAX3* analogue. As far as *Sox10* is concerned, quite apart from its significance in conserving survival [179] and multipotency of NCCs at the early stages of embryogenesis in mice [180], it is later crucial for melanocytic specification in zebrafish (*sox10*) [181] and prevention of premature neuronal differentiation of NCCs in mice [182], thus revealing its role in the WS phenotype. *MITF* mutations in humans are associated with WS2, and *MITF*’s role in the formation of osteoclasts, apart from being a key gene in the differentiation of NCCs into melanocytes, could explain the myoskeletal defects that are present in WS2 [183]. *EDN3/EDNRB* alterations have been linked with WS4 and, in some cases, with WS2. This binding molecule and its receptor are necessary for the maintenance, multiplication, and migration of NCCs that are going to differentiate into melanoblasts in mice [182]; they are also necessary for the hindrance of early differentiation of NCCs into enteric neurons in chick embryos [184]. Consequently, taking into consideration the roles of *EDN3/EDNRB* during neural crest differentiation, the phenotype of WS4 featuring pigmentation defects and HSCR can be justified. Finally, in some isolated WS cases, mutations in *SNAI2*—a promoter of the EMT process and a basic suppressor of cadherin interactions in neural crest cells—have been found. Its participation in the formation of embryonic mesoderm and various progenitors from NCCs, including melanocytic precursors [185], can explain the presence of its mutant in minor cases of WS2 [186,187].

#### 3.3.2. Piebaldism

Piebaldism is a disorder that is inherited in an autosomal dominant way, and it is characterized by pigmentation abnormalities. Specifically, its phenotype includes isolated vitiligo, which is distributed in the central scalp and forehead as well as in the middle portion of limbs and in the anterior part of central trunk. It is also associated with loss of hair color in the central forehead (poliosis). The key gene whose mutations are in charge of such pigmentation defects in the majority of collected clinical cases is *KIT* [188,189], with its role in the terminal differentiation of melanocytes in embryonic development being undoubtedly crucial. Melanocytes are formed by the differentiation of NCCs through a process mediated by KIT signaling. Ιn mice embryos, *Kit* acts synergistically with *Mitf*, a gene marker that is expressed in the pigmented retina epithelium, only in NCCs that are going to give rise to melanocyte precursors. This interaction determines the extent of the proliferation of melanocyte precursors. *Kit* is usually detected, apart from in melanocytes (where it is necessary for melanoblast migration), in cell populations like blood cells and primordial germ cells [178]. Another gene that has been proven to be involved in the pathogenesis of this disorder in some cases is *SNAI2*. Patients with Piebaldism who lacked mutations in *KIT* have been found with heterozygous deletion in *SNAI2*, a result that is in accordance with the depigmentation that is observed in mice mutants for *Slug* (equivalent to *SNAI2* in humans) [190]. In Xenopus embryos, *snai2* is directly induced and stabilized by the WNT signaling pathway, an interaction that in zebrafish is necessary for the differentiation of cranial NCCs into melanocytes, suggesting a possible explanation for pigmentation defects resulting from *wnt/snai2* mutations [191].

#### 3.3.3. Oculocutaneous Albinism (OCA)

OCA is in the category of rare congenital diseases that affect the production of melanin from melanocytes, products of NCCs. Melanin is responsible for pigmentation. The basic phenotype includes decreased melanin levels in the skin, hair, and iris, along with ocular abnormalities like nystagmus and decreased visual sharpness. Patients with OCA are more sensitive to skin damage caused by sun and have an increased risk of skin malignancies [192], as the pigment that is missing has a protective role against skin damage caused by UVR [193]. It should be noted that people with albinism have a proper population of melanocytes, but they are defective due to a mutation in a gene crucial for melanogenesis. The responsible gene is *TYR*, which is necessary for melanin formation. Specifically, melanoblasts come from the differentiation of NCCs during embryogenesis. After the process of EMT and the induction of migration, melanoblast precursors express *MITF*, a transcription factor that activates a variety of other genes involved in pigmentation; these include *TYR*, which converts L-tyrosine into DOPA within the pathway of melanin production [194]. Another gene involved in the proper differentiation of NCCs into melanoblasts is *KIT*, which increases the viability of melanoblasts and prevents their programmed cell death [195]. The WNT/β-catenin pathway is also present in melanogenesis as it is responsible for determining melanoblasts’ melanocytic fate after their terminal differentiation [196]. NOTCH signaling is also significant for pigmentation as it takes part in the development and survival of melanocytes. Its absence results in gray hair in mouse experiments, as the melanocytic population directly affects the amount of melanin produced [197]. Disruptions at any of these stages of the pigmentation procedure are responsible for the hypocolorization observed in OCA.

#### 3.3.4. Congenital Central Hypoventilation Syndrome (CCHS)

CCHS, otherwise known as Ondine’s curse, is an uncommon congenital disorder with an autosomal dominant inheritance. It is a life-threatening condition that affects ventilation because of the abnormal development of the autonomic nervous system [198]. The gene that is responsible for CCHS is *PHOX2B*, and its mutations are separated into two categories; polyalanine repeat expansion mutations (PARMs) and non-PARMs (NPARMs) [199]. In mice embryos, the presence of the transcription factor *Phox2b* is crucial for the sustenance of sympathetic precursors, and its expression is triggered by BMP signaling. *Alk3*, the receptor of BMP, controls the regulation of *Phox2b*, and the absence of *Phox2b* might explain the absence of sympathetic nervous system precursors in *Alk3* knockout embryos [200]. Its contribution to the development of the mouse’s sympathetic nervous system is achieved by leading neural progenitors that arise from the neural crest to stop proliferating and start differentiating into neurons [201]. *Phox2b* promotes the formation of motor neurons during embryogenesis, while after birth, it is distributed in the medulla (together with medullary cells that are present in the adrenal glands) and the pons [201,202,203]. Along with *Phox2a*, they are present in the autonomic ganglia and sensory ganglia of the skull [204], with *Phox2b* also being responsible for the development of the carotid body and other chemoreceptors that regulate the function of the ventilation center by recognizing levels of carbon dioxide [205]. Consequently, loss of *Phox2b* in mice means that they will not be able to form autonomic ganglia and sensory cranial ganglia properly, with the last of them being responsible for autonomic reflexes [204]. Apart from malformation of autonomic ganglia, knockout mice are characterized by increased apoptosis and lack of TH and DBH, revealing the role of *Phox2b* in the determination of neurotransmitter identity [204,206]. In humans, the mechanism that causes CCHS seems to be common with that of the mice, leading to a phenotype that involves inadequate ventilation, mainly during their sleep at the NREM stage, because of dysregulation of autonomic nervous system; this is because *PHOX2B* mutations affect both the autonomic ganglia and the formation of chemoreceptors [202]. In one rare case, a patient was recorded as having CCHS but no mutations in *PHOXB2*. Mutations were instead in the *RET* gene, which plays a role in sensitivity to the amount of inhaled carbon dioxide. Isolated mutations in other genes like *EDN3*, *BMP2*, *MYO1H*, *LBX1* have also been mentioned but are not usually encountered in the majority of cases of CCHS [207,208,209] pathogenesis.

### 3.4. Enteric–Sacral NCPs

#### Hirschsprung Disease (HSCR)

HSCR is a congenital disorder identified by the lack of inherent ganglion cells in the myenteric and submucosal wall plexuses/layers of the lower gastrointestinal tract, causing irregularities in smooth muscle function and leading to absence of gut motility and intestinal blockage [209]. The most common gene that is altered and causes lack of enteric ganglia (and consequently HSCR) is *RET*. In humans, *RET* is important during embryogenesis for the multiplication of enteric NCCs before the neural progenitors start to form neurons, as it upregulates the expression of genes. Mutations in its sequence, as a result, cause a reduction in the number of enteric crest cells in the developing gut, leading to a complete deficit in the enteric nervous system, a situation that is also confirmed in mouse embryos. Additionally, in humans, *RET* cooperates with *EDNRB*, which results in a transcriptional dependence between them, meaning that *EDNRB* can also be involved in the pathogenesis of HSCR. GDNF, a ligand that is responsible for the activation of *RET*, has a similar dependence [210]. Another gene involved in embryonic development that can be correlated with HSCR is *BMP4*. In chicken embryos, during embryogenesis, *BMP4* regulates migration of enteric NCCs and their terminal differentiation into glial cells or neurons, with its inhibition leading to the formation of neurons rather than ganglia [211,212]. In mutants that lack *BMP4*, the impaired ability to form enteric ganglia leads to hypoganglionosis of the hindgut [213]. An additional gene involved in pathogenesis of this disease is *PAX3*. In mouse embryos, *Pax3*, together with *Sox10* and *c-Ret*, plays a vital role in the development of the neural crest in the intestine and the enteric ganglia in particular, the malformation of which can lead to HSCR. Precursor cells that express *Pax3* are fated to form enteric ganglia, a morphogenesis event that *Pax3* is crucial for [214]. Mutations in *Pax3* could, consequently, explain the absence of gut innervation and aganglionosis that are characteristics of HSCR. *SOX10* is also a gene of interest for HSCR. In Xenopus embryos, *sox10* is necessary for both the survival and the induction of the terminal differentiation of progenitor cells that come from the neural crest, which later give rise to the melanocytes and ganglia of the peripheral nervous system. Mutants that are depleted of this gene are characterized by a loss of expression of marker genes *Mitf* and *c-kit*, which are specially expressed in melanocytic lineage, and c-ret, which is expressed in ganglia precursors, indicating the contribution of *sox10* to the absence of enteric ganglia in HSCR [215]. A number of other genes have been found to contribute to HSCR; these are *PHOX2B*, *L1CAM*, *NRG1*, *SIP1*, *GFRA1*, *ECE1*, *NTN*, *ARTN*, and *PSPN* [216,217,218,219,220,221,222]. All the aforementioned NCPs and implicated genes are summarized in Table 1.

## 4. Cancers

### 4.1. Melanocytic Cancers

#### Malignant Melanoma

Malignant melanoma is the most common cancer originating from NCCs. It is caused by the uncontrolled proliferation of melanocytes that are responsible for the coloration of the skin [223]. Melanomas metastasize aggressively, possibly due to melanocyte expression of EMT factors, and they have a poor prognosis. Animal models suggest melanoma cell lines follow NCC migration pathways. In about 50% of melanoma cases, activated mutations in *BRAF* gene are identified, while other cases include variants that play a significant role in the process of embryogenesis and are necessary not only for the proper differentiation of NCCs into melanocytes but also for the EMT phase. Canonical WNT signaling, together with the TGF-β/BMP signaling pathways, is of great significance for the induction of the EMT process during embryogenesis in many species [224,225] because it alters the interactions and adhesion between them. At the same time, these pathways are crucial for the pathogenesis of melanoma, because, in a similar way to EMT, they alter interactions between neoplastic melanocytes. The exact impact of these signaling pathways in the progression of the disease is yet unclear. Research conducted in human metastatic melanoma cell lines indicates that β-catenin upregulates both migration and invasion of neoplastic cells through the EMT procedure that is a milestone of embyogenesis in normal NCCs [225]. However, on the other hand, a survey including zebrafish embryos concluded that the WNT or TGF-β/BMP pathways facilitated the regeneration of melanocytes but strongly inhibited the invasiveness, migration, and proliferation of human melanoma cells [223]. Additionally, *KIT* is a gene that leads one of the basic pathways for melanoblast migration after their differentiation from NCCs. Although its exact mechanism is not yet fully understood, it acts synergistically with *MITF* (another gene that orchestrates the normal pigmentation process) and determines the extent of the proliferation of melanocyte precursors [178].

In some melanoma cases, mutations in *KIT* have been detected more frequently in the acral, mucosal, and chronically sun-damaged skin, a fact that is in accordance with the places that KIT-mutated melanoma first appears [226,227]. As far as *MITF* is concerned, in mouse embryos, it is a transcription factor that is specific and indicative of melanocyte progenitors. This marker gene is responsible not only for the survival of melanocyte progenitors by regulating anti apoptotic factors (ex. Bcl12) but also for cell multiplication and cell differentiation [228]. Its roles are preserved in neoplastic melanocytes, which maintain reliance on *MITF* [229]. Melanocytes that exhibit an increase of *MITF* expression have the capacity to either differentiate or multiplicate. Conversely, reduced *MITF* activity is associated with invasive behavior [230]. *PAX3* is another gene involved both in embryogenesis and melanoma. It belongs to the PAX family of genes, whose expression is predominantly noticeable in embryonic development, but it becomes inactive as most structures derived from NCCs undergo final differentiation in later stages [60]. Although it plays a crucial role in the development and viability of melanoblasts during embryonic stages [231,232], its expression extends to melanocyte stem cells and pluripotent precursor cells derived from the skin in post-embryonic stages. In stem cells, it seems to inhibit terminal differentiation, and, alongside its antiapoptotic capacity, it encourages the commitment of cells originating from neural crest to the melanocyte lineage [60,233]. In melanomas, *PAX3* is associated with non-chronic sun-damaged tumors and with tumors that are caused by chronic exposure to the sun, with the latter having the ability to affect molecules included in the *PAX3* pathway and lead to its loss of function [234]. Other genes involved in melanoma are *NRAS* [235] and *NF1* [236].

### 4.2. Schwann Cell Cancers

#### Schwannoma

Schwannomas are tumors of the peripheral nervous system that are most common in adults and consist of Schwann cells and other local cell types that partake in the creation of the neoplastic microenvironment [237]. Schwannomas are more prone to appear in areas affected by trauma and strain. *NF2* and *SOX10* are the identified genes related to the genesis of these tumors. The *NF2* tumor suppressor gene has been implicated in Schwann cells’ tumorigenic transformation, and its loss of function can lead to schwannomas [237,238,239]. *SOX10* is a characteristic neural crest gene that is detected in glial cell progenitors [240] and is a major regulator of peripheral glial development [241]. It drives some forms of segmental schwannoma, particularly schwannomas arising from non-vestibular cranial nerves [242,243]. *SOX10* encodes a transcription factor that regulates the differentiation and myelination of Schwann cells [243]. Furthermore, in the embryo, *SOX10* is a crucial gene that is responsible for the differentiation of NCCs into neuronal and glial progenitors, while it also plays a substantial role in the formation of enteric ganglia [244]. SOX10’s significance in the assumption of fate and differentiation in neuronal lineages support its involvement in the pathogenesis of schwannomas. Another hypothesis that has been made is that Hedgehog signaling might contribute to schwannomas’ formation, but further testing and research is required.

### 4.3. Sympathetic Cell Cancers

#### 4.3.1. Neuroblastoma

Neuroblastoma is the most common tumor in infancy and the most common tumor of extracranial location in childhood. It arises from stem cells of the sympathetic nervous system and especially sympathetic ganglia, which are composed of cells of NCC origin [245]. The vast majority of neuroblastomas exhibit mutations in *PHOX2B*, which is a neural crest gene involved in the formation of the peripheral nervous system [246]. In the embryo, the initiation of sympathetic neuron differentiation is characterized by BMPs production then followed by the induction of multiple transcription factors such as *Phox2b*. Research in chick embryos unveiled a step-by-step initiation of gene activity, commencing with *Phox2b* and *Ascl1* and succeeded by other transcription factors [247]. The presence of the transcription factor *Phox2b* is crucial for the sustenance of sympathetic precursors. As *Phox2b* expression is triggered by BMP signaling, *Alk3*, the receptor of *Bmp*, controls the regulation of *Phox2b*, and the absence of *Phox2b* might explain the absence of sympathetic nervous system precursors in *Alk3* knockout embryos [200]. Its contribution to the development of the mouse’s sympathetic nervous system is achieved by guiding neural progenitors that arise from neural crest in order for them to start differentiating into neurons [180]. Loss-of-function mutations in *PHOX2B* have been demonstrated to hinder the differentiation of neuroblastoma by impeding the maturation of early sympathetic neurons [248]. As a result, the crucial role of *PHOX2B* in sympathetic ganglia formation can be used to outline the basics of neuroblastoma pathogenesis. Unrelated to the neural crest, other genes implicated in neuroblastoma are *MYCN*, *ALK*, *ATRX*, and *TERT* [249].

#### 4.3.2. Pheochromocytomas–Paragangliomas (PPGLs)

PPGLs are uncommon tumors, in their majority benign, whose origins are chromaffin cells that originate from NCCs. They can be categorized either as sporadic types or those that develop on a genetic basis, with inherited paraganglioma being more frequent than the inheritable type of pheochromocytoma [250]. They are otherwise known as neuroendocrine tumors. Common symptoms of these neoplasms include increased blood pressure in combination with headaches, sweating, and a sensation of rapid, irregular, or strong heartbeats [251]. Questions have previously been raised about the contribution of *PHOX2B* mutations to the etiopathogenesis of these tumors. As has been previously explained, *PHOX2B* is the master gene in the development of the autonomic nervous system from NCCs during embryogenesis, which controls the expression of DBH and TH from the medulla of adrenal glands in mice [204]. So, mutations in *PHOX2B* could explain dysregulations in the development of the sympathetic nervous system, resulting in alterations in the level of catecholamines and therefore the phenotype of PPGL featuring hypertension and palpitation. Nowadays, it is estimated that around 40% of PPGLs are positive in *PHOX2B* mutations [252]. Another gene whose variants are responsible for PPGL is *SDHB*, which is also associated with the type of the cancer that is more likely to metastasize [253]. Another rare mutation that is linked with PPGL is that in the *RET* proto-oncogene, which also plays a significant role in the proliferation of enteric neural crest cells and the formation of enteric ganglia (see HSCR) [232]. *SDHC*, *SDHD*, and *VHL* are some other variants that have been identified in rare cases with these neoplasms [248,254].

### 4.4. Cancers from Multiple Lineages

#### Familial Medullary Thyroid Carcinomas

Familial medullary thyroid carcinoma is a malignant neural crest-derived cell tumor, which concerns the calcitonin-secreting parafollicular C cells of the thyroid gland. It can be sporadic or related to multiple endocrine neoplasia type 2 syndrome [255]. *RET* mutations are the predominant cause of pathogenesis of this tumor [256]. *RET* is a proto-oncogene that produces a tyrosine kinase receptor that is notably present in neural crest-derived cells, including the parafollicular C cells of the thyroid, and is crucial for controlling cell proliferation, migration, and terminal differentiation of neural crest-derived tissues [257]. Medullary thyroid carcinomas have been found to overexpress proto-ret mRNA [258], indicating *RET* overexpression as one of the main players in the tumor’s pathophysiology. *PITX2*, which is expressed in NCCs, is also involved in the genesis of the tumor and is implicated in WNT/b-catenin signaling [255]. All the aforementioned cancer-based NCPs and implicated genes are summarized in Table 2.

## 5. Conclusions and Future Perspectives

Recognizing the uniqueness of each patient, health practitioners have traditionally relied on generalized one-size-fits-all approaches due to limited understanding of individual differences. However, with the rise of “personalized medicine”, these conventional methods are expected to become outdated [259]. Although precision medicine is widely accepted in many fields, its adoption in reproductive medicine is still in its early stages. Despite advancements in in vitro fertilization (IVF) techniques over the past forty years, success rates remain relatively low at 25–30% per cycle. New technologies such as next-generation sequencing (NGS), time-lapse imaging, and various omics approaches, combined with artificial intelligence, offer hope for a deeper understanding of biomarkers and improved clinical outcomes tailored to each patient [260].

Interestingly, studies investigating the developmental signaling pathways regulating neural crest cell migration and differentiation have identified key molecular players that are dysregulated in NCPs. This knowledge assists in developing precision medicine approaches that specifically target the underlying molecular defects in individual patients [261,262]. Emerging technologies such as induced pluripotent stem cells (iPSCs) offer a revolutionary platform for modelling NCPs in the laboratory. Researchers can study disease mechanisms, screen potential therapeutics, and develop personalized treatment approaches by reprogramming patient-specific cells into neural crest derivatives. This innovative use of iPSCs bridges the gap between embryological insights and precision medicine, paving the way for more targeted and effective interventions in NCPs [263].

The second critical application of precision medicine in NCPs is identifying genetic variants associated with these conditions. Through whole-exome or genome sequencing and understanding the precise role of each gene in embryonic development, researchers can pinpoint the specific genetic mutations that predispose individuals to NCPs, enabling diagnosis and targeted interventions as early as possible [264]. By analyzing the genetic landscape of individual patients, healthcare providers can predict disease progression, assess the risk of complications, and design personalized treatment plans. In conclusion, integrating embryological insights with precision medicine in NCPs opens new avenues for diagnosis and treatment.

## Figures and Tables

**Table 1:** Neurocristopathies and potential gene targeting.

Affected Regions	Accepted Examples	Genes	References
Cranial–Facial Regions	Goldenhar syndrome	*MSX1*, *MYT1*, *SF3B2*	[41,42,46]
	Axenfeld–Rieger syndrome	*FOXC1*, *FOXC2*, *PITX2*, *CYP1B1*, *PRDM5*, *JAG1*, *USP9X*, *CDK13*, *HCCS*, *AMELX*, *BCOR*	[49,50,53]
	Craniosynostosis	*FGFR1*, *FGFR2*, *FGFR3*, *TWIST1*, *EFNB1*	[55]
	Craniofacial–deafness–hand syndrome	*PAX3*	[60]
	Tricho-dento-osseus syndrome	*DLX3*	[65]
	Peter’s anomaly	*PITX2*, *PAX6*, *FOXE3*, *FOXC1*, *CYP1B1*	[63,64,68,70]
	Bamforth–Lazarus syndrome	*FOXE1*, *MSX1*, *TGF-b3*	[53,73]
	Branchio-oculo-facial syndrome	*TFAP2A*	[79]
	CADASIL	*NOTCH3*, *FOXC1*, *PITX2*, *TIMP3*, *VTN*	[86,94,95]
	Congenital aniridia	*PAX6*, *FOXC1*, *PITX2*, *TRIM44*, *ELP4*, *DCDC1*, *CYP1B1*	[96,99,100]
	Frontonasal dysplasia	*ALX3*, *ALX4* *ALX1*, *EFNB1*, *KIF3A*	[101,103,104,111,112]
	Hajdu–Cheney syndrome	*NOTCH2*	[113]
	Moebius syndrome	*PLXND1*, *REV3L*, *TUBB3*, *HOXA1*, *HOXB1*, *GSH1*, *CDX2*, *CRBP1*, *PBX2*, *EGR2*, *SOX14*	[21,126,127,129,131,132,133,134,135]
	Pierre Robin sequence	*SOX9*, *BMP2*	[137]
	Mowat–Wilson syndrome	*ZEB2*	[146]
	SAMS disorder	*GSC*, *EDNRA*	[150,154]
Heart and Outflow Tract	DiGeorge syndrome	*TBX1*, *GBX2*, *CXCR4*, *CXCL12*	[77,158]
	CHARGE syndrome	*CHD7*, *TBX1*	[117]
	Velocardiofacial syndrome	*TBX1*	[77]
	Fetal alcohol spectrum disorders	*SHH*	[163]
	Alagille syndrome	*JAG1*, *NOTCH2*	[117]
	Noonan–LEOPARD syndrome	*PTPN11*, *SOS1*, *KRAS*, *NRAS*, *RAF1*, *BRAF*, *SHOC2*, *MAP2K1*, *CBL*	[53,171]
	Jacobsen syndrome	*ETS1*	[174]
Melanocytes and Neuronal Ganglia	Piebaldism	*KIT*, *SNAI2*	[188,189,190]
	Waardenburg syndrome	*PAX3*, *EDN3*, *EDNRB*, *SOX10*, *MITF*, *SNAI2*	[178,181,182,183,186,187]
	Oculocutaneous albinism	*TYR*, *KIT*	[194,195]
	Congenital central hypoventilation syndrome	*PHOX2A*, *PHOX2B*, *GDNF*, *RET*, *EDN3*, *BMP2*, *MYO1H*, *LBX1*	[199,207,208,209]
Enteric–Sacral Nervous System	Hirschsprung disease	*RET*, *EDNRB*, *BMP4*, *PAX3*, *L1CAM*, *NRG1*, *SOX10*, *SIP1*, *PHOX2B*, *GFRA1*, *HASH1*, *HAND2*, *ECE1*, *GDNF*, *NTN*, *ARTN*, *PSPN*	[210,211,214,215,216,217,218,219,220,221,222]

**Table 2:** Cancer-based neurocristopathies and genetic correlations.

Affected Structures	Types of Cancer	Genes	References
Cranial–Facial Regions	-	-	-
Heart And Outflow Tract	Familial medullary thyroid carcinomas	*RET*, *PITX2*	[255,256]
Melanocytes, Neuronal Ganglia, and Nerves	Malignant melanoma	*KIT*, *PAX3*, *MITF*, *BRAF*, *NRAS*, *NF1*, *CDKN2A*, *CDK4*	[60,178,226,227,229,233,235,236]
	Schwannoma	*SOX10*, *NF2*	[237,238,239]
	Neuroblastoma	*PHOX2B*	[246,249]
	Pheochromocytoma–Paraganglioma	*PHOX2B*, *SDHB*, *SDHC*, *SDHD*, *VHL*	[248,252,254]
Enteric Nervous System	-	-	
Sacral	-	-	

## Abbreviations

ALK	ALK receptor tyrosine kinase
ALX	ALX homeobox
AMELX	Amelogenin X-linked
ARS	Axenfeld–Rieger Syndrome
ARTN	Artemin
ATRX	ATRX chromatin remodeler
B3GLCT	Beta 3-glucosyltransferase
BCOR	BCL6 corepressor
BLS	Bamborth–Lazarus Syndrome
BMP	Bone morphogenetic protein
BOFS	Branchio-oculo-facial syndrome
BRAF	B-Raf proto-oncogene
CADASIL	Cerebral autosomal dominant arteriopathy with subcortical infarcts and leukoencephalopathy
CCHS	Congenital central hypoventilation syndrome
CDH	Cadherin
CDK13	Cyclin-dependent kinase 13
CDX2	Caudal type homeobox 2
CFDS	Craniofacial–deafness–hand syndrome
CFNS	Craniofrontonasal syndrome
CHD7	Chromodomain helicase DNA-binding protein 7
COL	Collagen
CRBP1	Retinol-binding protein 1
CTF	C terminal fragment
CTF	C-Terminal Fragment
Cxcl12	C-X-C motif chemokine ligand 12
Cxcr4	C-X-C motif chemokine receptor 4
CYP1B1	Cytochrome P450 family 1 subfamily B member 1
DBH	Dopamine beta-hydroxylase
DCDC1	Doublecortin domain-containing 1
Dkk	Dickkopf WNT signaling pathway inhibitor
DLX	Distal-less homeobox
DOPA	Dihydroxyphenylalanine
ECE1	Endothelin-converting enzyme 1
ECG	Electrocardiography
EDN3	Endothelin 3
EDNRB	Endothelin receptor type B
EFNB1	Ephrin-B1
EGF	Epidermal growth factor
EGR2	Early growth response 2
ELP4	Elongator acetyltransferase complex subunit 4
EMT	Epithelial–mesenchymal transition
ETS1	ETS proto-oncogene 1, transcription factor
FGF	Fibroblast growth factor
FGFR	Fibroblast growth factor receptor
FND	Frontonasal dysplasia
FOXC1	Forkhead box C1
FOXD3	Forkhead box D3
FOXF	Forkhead box F
FOXI1/2	Forkhead box I1/2
GATA	GATA binding domain
GBX2	Gastrulation brain homeobox 2
GFRA1	Glial cell-derived neurotrophic factor family receptor alpha 1
GLI1	GLI family zinc finger 1
GSC	Goosecoid
GSX1	GS homeobox 1
HCCS	Holocytochrome c synthase
HCS	Hajdu–Cheney syndrome
HOX	Homeobox
HSCR	Hirschsprung disease
iPSCs	Induced pluripotent stem cells
IVF	In vitro fertilization
JAG1	Jagged canonical Notch ligand 1
KIT	KIT proto-oncogene, receptor tyrosine kinase
KRAS	KRAS proto-oncogene, GTPase
L1CAM	L1 cell adhesion molecule
LBX1	Ladybird homeobox 1
MAP2K1	Mitogen-activated protein kinase kinase 1
MAPK	Mitogen-activated protein kinase 1
MBS	Moebius syndrome
MITF	Melanocyte-inducing transcription factor
MSX	Msh Homeobox
MWS	Mowat–Wilson syndrome
MYCN	MYCN proto-oncogene
MYO1H	Myosin IH
MYT1	Myelin transcription factor 1
NC	Neural crest
NCCs	Neural crest cells
NF1	Neurofibromin 1
NGS	Next-generation sequencing
NOTCH	NOTCH receptor
NRAS	NRAS proto-oncogene
NRAS	NRAS proto-oncogene, GTPase
NREM	Non-rapid eye movement
NRG1	Neuregulin 1
NSD	Nuclear receptor binding SET domain protein
NSD3	Nuclear receptor binding SET domain protein 3
NTN1	Netrin 1
OAVS	Oculo-auriculo-vertebral spectrum
OCA	Oculocutaneous albinism
PA	Pharyngeal arch
PARMs	Polyalanine repeat expansion mutations
PAX3/6/7	Paired Box3/6/7
PBX2	Pre-B-cell leukemia homeobox 2
PHOX2B	Paired-like homeobox 2B
PITX2	Paired-like homeodomain 2
PPGL	Pheochromocytoma–paraganglioma
PRDM5/16	PR/SET domain 5/16
PRS	Pierre Robin sequence
PSPN	Persephin
PTPN11	Protein tyrosine phosphatase non-receptor type 11
RAB	Member of the RAS oncogene family
RAF1	Raf-1 proto-oncogene, serine/threonine kinase
RET	Rearranged during transfection
ROBO	Roundabout guidance receptor
SAMS	Short stature, auditory canal atresia, mandibular hypoplasia, and skeletal abnormalities
SDHB	Succinate dehydrogenase complex iron sulfur subunit B
SDHC	Succinate dehydrogenase complex subunit C
SDHD	Succinate dehydrogenase complex subunit D
SF3B2	Splicing factor 3b subunit 2
SHH	Sonic hedgehog
SHOC2	SHOC2 leucine-rich repeat scaffold
SIP1	Smad interaction protein 1
SLIT	Slit guidance ligand
SNAI1/2	Snail family transcriptional repressor 1/2
SOS1	Son of Sevenless Ras/Rac guanine nucleotide exchange factor 1
SOX	SRY-box transcription factor
SP7	Transcription factor Sp7
Tak1	Transforming growth factor beta-activated kinase 1
TBX1	T-box transcription factor 1
TDO	Tricho-dento-osseous syndrome
TERT	Telomerase reverse transcriptase
TFAP2	Transcription factor AP-2
TGF-β	Transforming growth factor beta
TH	Tyrosine hydroxylase
TIMP3	Tissue inhibitor 3 of metalloprotease-1
TRIM44	Tripartite motif-containing 44
TUBB3	Tubulin beta 3 class II
TWIST	Twist family bHLH transcription factor
USP9X	Ubiquitin specific peptidase 9 X-linked
UVA	Ultraviolet A
VHL	Von Hippel–Lindau tumor suppressor
VSMCs	Vascular smooth muscle cells
WS	Waardenburg syndrome
ZEB2	Zinc finger E-box binding homeobox 2
ZIC1	Zic family member 1
